# Anti-HIV Activity Mediated by Natural Killer and CD8+ Cells after Toll-Like Receptor 7/8 Triggering

**DOI:** 10.1371/journal.pone.0001999

**Published:** 2008-04-23

**Authors:** Erika Schlaepfer, Roberto F. Speck

**Affiliations:** Division of Infectious Diseases and Hospital Epidemiology, Department of Internal Medicine, University Hospital of Zurich, Zurich, Switzerland; Institut Pasteur Korea, Republic of Korea

## Abstract

We previously found that triggering TLR7/8 either by single stranded HIV RNA or synthetic compounds induced changes in the lymphoid microenvironment unfavorable to HIV. In this study, we used selective TLR7 and 8 agonists to dissect their contribution to the anti-HIV effects. While triggering TLR7 inhibited efficiently HIV replication in lymphoid suspension cells from tonsillar origin, its effect was inconsistent in peripheral blood mononuclear cells (PBMC). In contrast, triggering TLR8 showed a very prominent and overall very consistent effect in PBMC and tonsillar lymphoid suspension cells. Depletion of dendritic cells (DC), Natural killer cells (NK) and CD8+ T-cells from PBMC resulted in the reversal of TLR8 induced anti-HIV effects. Especially noteworthy, depletion of either NK or CD8+ T-cells alone was only partially effective. We interpret these findings that DC are the initiator of complex changes in the microenvironment that culminates in the anti-HIV active NK and CD8+ effector cells. The near lack of NK and the low number of CD8+ T-cells in tonsillar lymphoid suspension cells may explain the lower TLR8 agonist's anti-HIV effects in that tissue. However, additional cell-type specific differences must exist since the TLR7 agonists had a very strong inhibitory effect in tonsillar lymphoid suspension cells. Separation of effector from the CD4+ target cells did not abolish the anti-HIV effects pointing to the critical role of soluble factors. Triggering TLR7 or 8 were accompanied by major changes in the cytokine milieu; however, it appeared that not a single soluble factor could be assigned for the potent effects.

These results delineate the complex effects of triggering TLR7/8 for an efficient antiviral defense. While the ultimate mechanism(s) remains unknown, the potent effects described may have therapeutic value for treating chronic viral diseases. Notably, HIV replication is blocked by TLR triggering before HIV integrates into the host chromosome which would prevent the establishment or maintenance of the latent reservoir.

## Introduction

Pattern recognition receptors act as sentinels against microorganisms [1]. They recognize conserved motifs of microorganisms and trigger distinct signaling pathways that result in coordinated cellular changes critical to the immediate innate defense and the generation of adaptive immune response.

Toll-like receptors (TLR) are an essential family of pattern recognition receptors [1]. They can be grouped by their preferences for structural motifs. TLR3, 7, 8, and 9 are implicated in anti-viral defense [1], [2]. TLR3 recognizes double-stranded viral RNA [3], TLR7 and 8 recognize single-stranded viral RNA [4], [5] and TLR9 unmethylated DNA rich in cytosine-guanosine motifs from bacteria and viruses [6]. TLR3 is expressed on myeloid dendritic cells (MDC), TLR7 and 9 on plasmacytoid dendritic cells (PDC), and TLR8 on MDC and monocytes [7], [8]. While the transduction pathways of individual TLRs are well described [9], the overall effects of TLR triggering on the lympho-reticular system and on viral diseases remain largely unknown.

Compounds triggering distinct TLR may be of great value for targeted immunomodulation of viral diseases, including HIV [10]. The complex changes from triggering TLR may be more beneficial than administration of single cytokines. In fact, human studies examining treatments with distinct cytokines have rather been discouraging [11]. A detailed mechanistic examination is certainly required for the targeted use of compounds triggering TLR.

We previously reported that the TLR7/8 agonist R-848 or HIV ssRNA has immediate anti-HIV activities [12]. Furthermore, TLR7/8 agonists which were given as vaccine adjuvants in concert with HIV proteins were able to impact on the magnitude and quality of anti-HIV CD8+ T-cell responses [13], [14]. Thus, triggering TLR7/8 stimulates the innate as well as the adaptive immune response; this unique property makes them, at least theoretically, superior to cytokines, which are conventionally given as single agent and either act on the innate or the adaptive arm of the immune system. Thus, triggering TLR7/8 may represent an ideal therapeutic strategy for immunomodulation stimulating multiple arms of the immune response.

In the previous work, we examined the synthetic compound R-848, which triggers TLR7 and 8. Since TLR7 is preferentially expressed on PDC [8], the major producer of IFN-***α***, TLR7 triggering is believed to be a key factor in the overall anti-viral response. However, IFN-***α*** did not have a major role in the anti-HIV activity that we observed since its neutralization did not reverse R-848's anti-HIV effect. To differentiate the individual contributions of triggering TLR7 and 8 to the anti-HIV activity requires agonists that specifically and efficiently trigger the corresponding TLR. Because TLR8 does not function in mice[15], this pathway has been less well studied, and its biologic significance is largely unknown.

In the present study, we used agonists that specifically trigger each pathway to characterize the individual contributions of TLR7 and 8 signaling to the overall anti-HIV effect. Our findings suggest novel strategies for anti-HIV therapies.

## Results

### Anti-HIV activity of triggering TLR7 and/or 8 depends on the origin of lymphoid tissue

**Figure 1:**
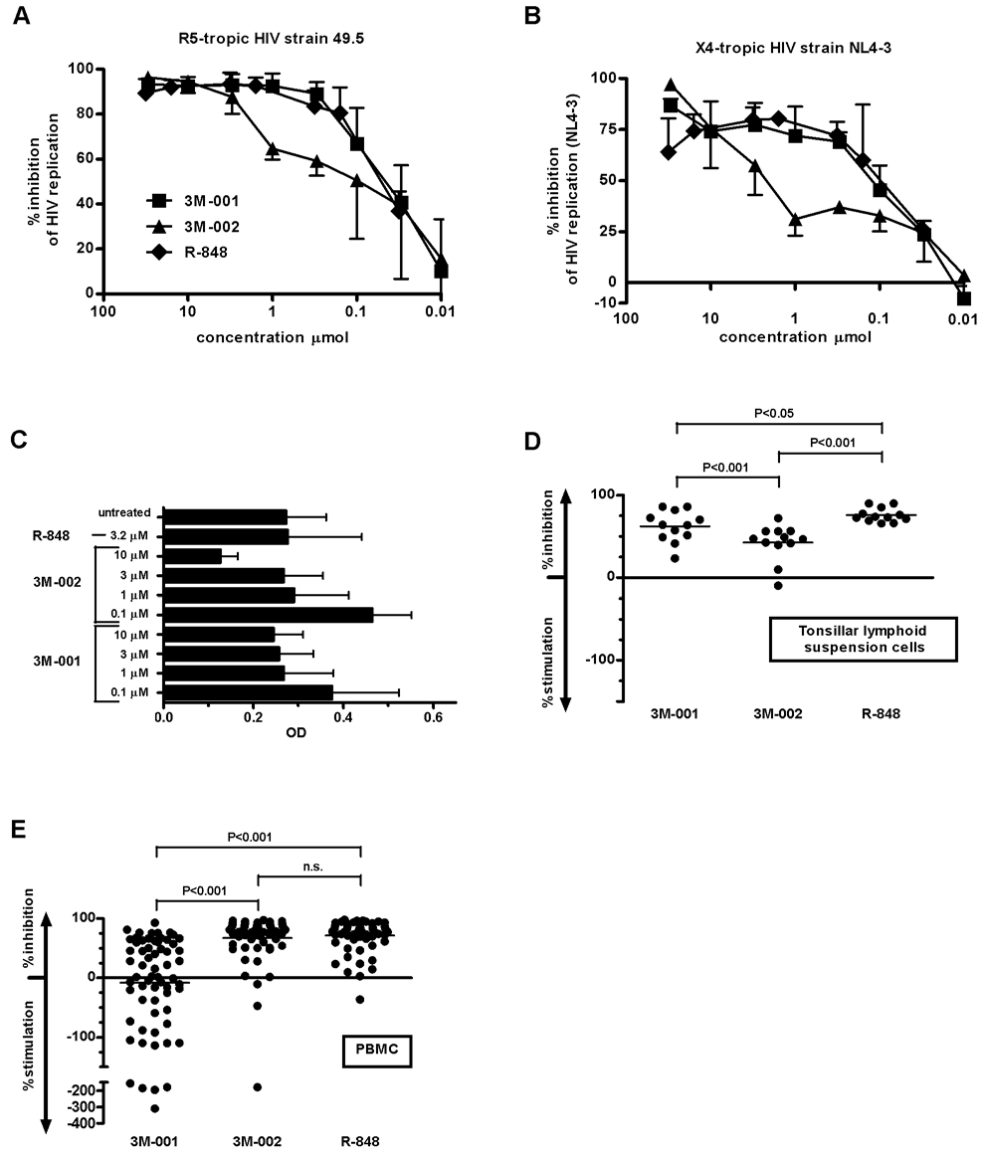
Triggering TLR7 and/or 8 results in strong anti-HIV activity that varied with the origin of human lymphatic tissue. Dose-dependent blocking of HIV by the compounds tested (▪ = 3M-001, ▴ = 3M-002, ♦ = R-848) when exposing tonsillar lymphoid suspension cells to (A) the R5-tropic strain 49.5 (n = 2) or (B) the X4-tropic strain NL4-3 (n = 6 for 3M-001 and 3M-002 and for data points 3, 10, 30 µm, otherwise n = 2). (C) No toxic effects were noted in lymphoid tissue at 3 µm of any of the TLR agonists, as measured by the WST-1 assay (n = 6). Anti-HIV activities (D) in tonsillar lymphoid suspension cells and (E) in PBMC. Data obtained with R-848 were partially published before [12]. Data were first analyzed by one-way analysis of variance and if tested significantly different, by Bonferroni's multiple comparison test.

We first determined the optimal working concentration of the TLR7 and/or 8 agonists; briefly, these agonists were added to tonsillar lymphoid suspensions cells in a range of 0.1–30 µmol; the tissue was infected two days later. HIV replication was monitored by measuring viral capsid p24 antigen (p24 Ag) in the supernatant. We also assessed the dose-dependent effects on cell viability of these TLR agonists. We found that the optimal working concentration was 3 µmol (Fig. 1A–C) which had a strong inhibitory effect on HIV replication while it had no negative effect on cell viability.

The HIV strains, NL4-3 (X4-tropic) and 49.5 (R5-tropic) we used replicates vigorously in untreated tonsillar lymphoid suspension cells and PBMC [12]. In tissue-matched tonsillar lymphoid suspension samples, triggering TLR7 or 7/8 resulted in substantial inhibition of NL4-3 replication, *i.e*., 3M-001: 64% (50.2 to 77.2) (median inhibition (25% to 75 percentile)); R-848: 73.5% (70.2 to 81.5)) (Fig. 1 D). Although less effective, triggering TLR8 still yielded an inhibition with a median of 46.9% (40.8 to 56.2). In contrast, in PBMC, triggering TLR8 resulted in substantially higher anti-HIV activity than triggering TLR7 (median inhibition 3M-001: 2.5% (−54 to 60.2) *vs* 79.5% (65.3 to 89)) (Fig. 1E). Not unexpected, the TLR7/8 agonist R-848 again had high levels of anti-HIV activity (median inhibition: 79.1% (87.1 to 69.3)). In other experiments, we verified that the changes in the microenvironment induced by TLR7 or 8 agonists had similar anti-HIV effects on a number of HIV strains with distinct co-receptor selectivity (anti-HIV activity subsequent to 3M-001 in tonsillar lymphoid suspension cell cultures infected with JR-CSF: 96.67% (96.42 to 96.72), 49.5: 93.5% (95.4 to 97.34), 89.6: 81% (75.6 to 86.4); anti-HIV activity subsequent to 3M-002: JR-CSF: 94.9% (94.7 to 95.2), 49.5: 91.4% (93.6 to 95.9), 89.6: 65% (73.7 to 82.5; n = 2; 49.5 and JR-CSF are R5-tropic strains, 89.6 is a dual-tropic strain).

The origin of the lymphoid tissue may be important for the distinct biologic effects observed. Indeed the cellular composition differed significantly between PBMC and tonsillar lymphoid suspension cells (Table 1). Most prominent was the nearly complete absence of NK and monocytes in tonsillar lymphoid suspension cell cultures. The percentage of CD4+, CD8+ and B-cells displayed also marked differences depending on the origin of the tissue. In contrast, the fraction of PDC and MDC was similar in PBMC and tonsillar lymphoid suspension cells.

**Table 1:** Differential cell count in PBMC and tonsillar lymphoid suspension cells in % (avg±std; n = 4).

	CD4+ cells	CD8+ cells	CD19+ cells (B-cells)	CD14+ cells (monocytes)	CD56+ cells (NK)	MDC	PDC
**PBMC**	37.2±7.5	19.0±7.5	13.3±4.4	6.3±2.4	10.6±3.0	0.5±0.4	0.8±0.7
**Tonsillar Lymphoid suspension cells**	19.5±1.5	3.8±1.1	67.0±7.2	0.6±0.27	0.3±0.07	0.5±0.09	1.0±0.58

### DC are critical for the anti-HIV effects observed

**Figure 2:**
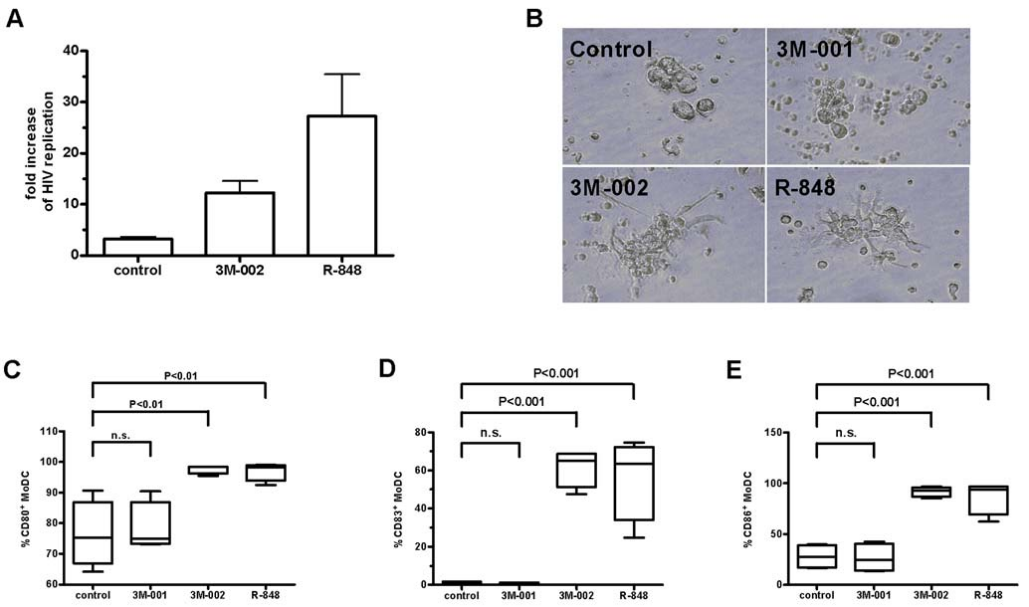
DC are critical in the generation of the microenvironment unfavorable for HIV replication. (A) Depletion of DC and DC precursors resulted in reversal of HIV inhibition (n = 2) despite triggering TLR8 and/or 7/8. Represented is the fold increase of HIV replication in PMBC depleted of DC as compared to matched samples. (B) The TLR agonists 3M-002 and R-848 which trigger TLR8 and 7/8 respectively resulted in clumping and activation of MoMDC. This was not the case with 3M-001, which triggers solely TLR7. (C–E) Triggering TLR8 or 7/8 resulted in marked activation of MoMDC as quantified by the cell-surface expression of CD80, CD83, and CD86 (n = 4). Data were first analyzed by one-way analysis of variance and if tested significantly different, by Bonferroni's multiple comparison test.

TLR7 and 8 show cell-specific expression with TLR7 expressed on B-cells and PDC, and TLR8 on monocytes and MDC [7], [8]. Based on their sentinel role and the expression of TLR8, we assumed that MDC are the most likely target cells responsible for the dramatic HIV inhibitory effects. Indeed when DC and precursor DC were depleted from PBMC, HIV replication increased 12-fold and 27-fold, despite adding the TLR8 and TLR7/8 agonists, respectively. Notably, we noted a 3-fold increase in lymphoid tissue lacking DC as compared to unfractionated lymphoid tissue; this 3-fold increase points to an antiviral action by DC triggered by HIV alone (Fig. 2A). We observed striking differences of cytokine released in the media between unfractionated PBMC and PBMC lacking DC indicating the successful depletion of DC (Table 2). The striking differences observed is explained by the lack of DC which produce cytokines [16] as well as by the lack of DC which will activate down-stream effector cells such as NK cells and CD8+ T-cells [17], [18]. Furthermore, there were differences in the cytokine release in response to the distinct TLR7 and/or 7/8 agonists tested. These results reflects the subset specific ability of DC to respond to TLR agonists [8] and underscores the importance of DC within the complex network of innate immunity. In line with data with R-848 [19], the potency of pure TLR8 agonists to activate monocyte-derived myeloid dendritic cells (MoMDC) was illustrated by their characteristic histological changes (Fig. 2B) and the marked increase of the maturation/activation markers, CD80, 83 and 86 one day after exposure to the TLR8 agonist 3M-002 (Fig. 2C–E). MoMDC which have been activated by TLR7 and/or 8 agonists show substantial induction of a large number of cytokines (Table 3).

**Table 2:** Measurement of cytokines (pg/ml) one day after triggering TLR7 and/or 8 in unfractionated PBMC and PBMC without DC (n = 2).

		control	3M-001	3M-002	R-848
**IFN-α**	Unfractionated PBMC	<8; 9.6	3624; 5950	1057; 499	1989; 4250
	PBMC without DC	<8; <8	<8; 53.8	9.6; 17.2	9.6; 37.2
**IL-12 (p40)**	Unfractionated PBMC	32; 19	1564; 1168	13608; 5777	12784; 5591
	PBMC without DC	101; 190	146; 56	185; 264	129; 190
**IL-15**	Unfractionated PBMC	<0.1; <0.1	0.59; 0.46	0.42; 0.21	0.52; 0.3
	PBMC without DC	<0.1; <0.1	<0.1; <0.1	<0.1; <0.1	0.1; <0.1
**IL-18**	Unfractionated PBMC	37; 16	46; 24	615; 590	253; 333
	PBMC without DC	<0.1; 1.3	<1; 1.8	6.1; 24.1	6.1; 15.4
**TNF-α**	Unfractionated PBMC	<0.9; <0.9	56; 101	37659; 33474	40332; 20478
	PBMC without DC	407; 1647	429; 727	586; 1397	56; 1482

All data presented in this table were measured by bead assay as described in the Material and Method section.

**Table 3:** Induction of cytokines (pg/ml) two days after triggering TLR7 and/or 8 in MoMDC; indicated are the average±std fold induction# and the individual absolute concentrations of cytokines measured in the supernatant [pg/ml] ¶.

	control	3M-001	3M-002	R-848
**IFN-γ**	0, 0.2, 0, 0¶	§		
		0,0,0.2,4.2¶	0.4, 0.8 , 0, 0	0, 0.2, 0, 0
**IL-6**	139, 150, 163, 100	1.1±0.3#	168±97	163±291
		209, 168, 168, 79¶	40000, 12650, 34078, 9318	83336, 1048, 6730, 496
**IL-8**	33558, 2708, 1117, 2199	1.1±0.7	73±73	15±13
		3094, 3963, 1132, 3558	98638, 125298, 195580, 146220	20395, 20675: 28866, 56422
**IL-12**	63, 299, 61, 85	1.8±1.5	1.5±1.1	1.5±0.8
		229, 5.4, 107,158	147, 69, 153, 71	123, 241, 147, 63
**TNF-α**	4.6, 0.2, 3.4, 10	4.0±3.3	511±913	99±124
		20.2,1.6,0.4,33.8	292, 376, 253, 261	233, 56, 196, 77
**MIP-1α**	45, 12, 45, 12	1.1±0.8	91±86	9.0±12
		12, 22, 32, 22	7085, 360, 184, 2034	133, 74, 22, 319
**MIP-1β**	609, 9.2, 49, 72	1.0±0.5	14±8.0	7.8±7.1
		277, 15, 49, 72	9633, 90, 298, 1811	1967, 123, 9.2, 1021
**RANTES**	8, 2.2, 8, 2.2	1.1±0.5	33.17±28.44	14.26±16.62
		6, 2.2, 6, 4	584, 23, 123, 75	36, 31, 6, 83

§ : induction can not be calculated since denominator is 0.

All data presented in this table were measured by enzyme immunoassay as described in the Material and Method section.

### NK and CD8 T cells are the main effector cells for the anti-HIV activity after triggering TLR7 and/or 8

**Figure 3:**
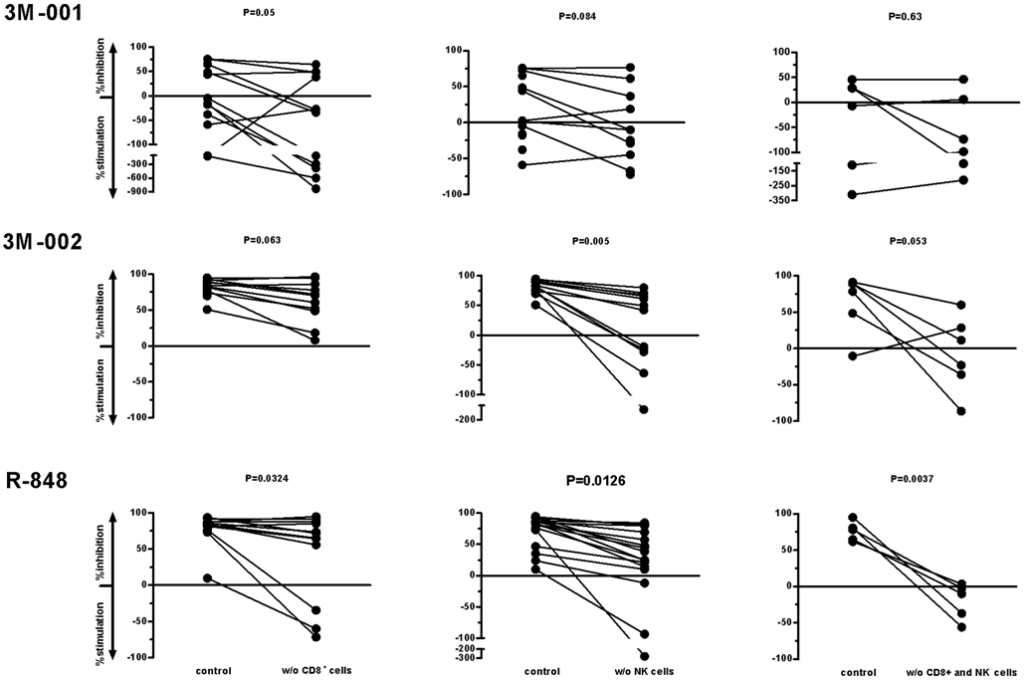
NK cells and CD8+ T-cells are the effector cells responsible for the anti-HIV effects in PBMC after triggering TLR8 or TLR7/8. PBMC from different donors were either depleted of CD8+ T-cells or NK or both, treated with the various TLR7 and/or 8 agonists and infected with the X4-tropic strain NL4-3 two days later. HIV infection was monitored by measuring p24 antigen in the supernatant over time. Statistical analysis was done by paired t-test.

We wanted to assess the role of CD8+ T-cells and NK cells for their significance in the TLR7/8's HIV inhibitory activity. For this purpose, we depleted these cell subsets by using microbeads, added the TLR7 and/or 8 agonists and infected the cells two days later. We found that depleting CD8+ T-cells reversed the TLR8 and/or 7/8s' anti-HIV effects minimally, that depleting NK cells were more efficient in that sense, but strikingly depleting both simultaneously resulted in a nearly complete reversal of the anti-HIV effects (Fig. 3).

**Figure 4:**
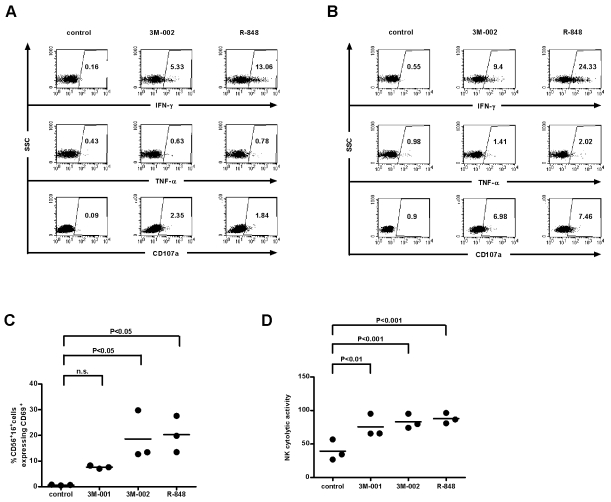
Triggering TLR8 or 7/8 results in strong activation of NK and CD8+ T-cells. PBMC were treated with the TLR agonists and 24 h later analyzed for intracellular IFN-γ, TNF-α and CD107a expression for CD8+ T-cells (A) and NK (B), for the expression of CD69 on NK cells (n = 3) (C) and for the NKs' cytolytic activity against K562 cells (n = 3) (D). Data were first analyzed by one-way analysis of variance and if tested significantly different, by Bonferroni's multiple comparison test.

Triggering TLR8 and/or 7/8 resulted in activation of CD8+ T-cells and NK cells, as assessed by expression of the activation marker CD69, by intracellular staining for IFN-γ and TNF-α, by measuring CD107a, and by their cytotoxic activity against the NK-responsive cell line K562 (Fig. 4). CD107a is a granular protein and is exposed at the cellular surface during degranulation of cytokines and of cytotoxic proteins such as perforin and granzyme [20]. Staining for CD107, thus, quantifies the cumulative cytolytic activity of a distinct cell population.

To characterize in depth the NK phenotype leading to the anti-HIV activity, we quantified a large number of NK-specific cell surface markers known for as activating or inhibitory receptors by flow-cytometry. In particular, we hypothesized that TLR7/8 triggering will favor NK to express activating and less inhibitory receptors. However, the expression of the activating NK receptor, NKG2D, was significantly lower subsequent to the exposure of PBMC to R-848 than in the control (control *vs* R-848: median (minimum–maximum) % of cells expressing NKG2D 2.9 (2.4–3.4) *vs* 1.42 (1.04–1.58), n = 3, p = 0.007, paired T-test). The levels of expression of other inhibitory (CD94, KIR2DL2, KIR3DL1, ILT2) and activating receptors (NKp30, NKp46, NKp44, DNAM-1) on NK cells were similar irrespective of triggering TLR7/8 (data not shown).

In contrast, triggering TLR7 and/or 7/8 strikingly up-regulated TRAIL (control vs 3M-001, 3M-002 and R-848: median (minimum–maximum) % of cells expressing TRAIL: 7.2 (3.8–14.2) *vs* 54 (34.3–75.2), 65 (44–73.3), 64 (37.9–79.4); ANOVA P = 0.003, P<0.05, <0.01 and <0.01, respectively).

### Soluble factors mediate anti-HIV effects after triggering of TLR7 and/or 8

**Figure 5:**
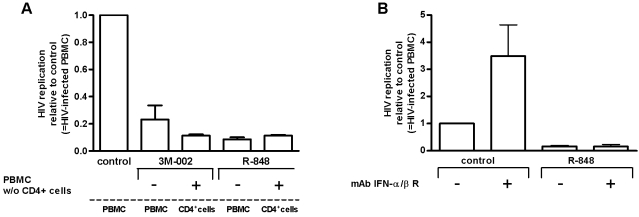
Soluble factors are critical to the anti-HIV effects after triggering TLR8 and 7/8. (A) Isolated CD4+ T-cells of PBMC were infected with HIV overnight. Transwells were added with the purpose to separate the HIV infected CD4+ T-cells by a semi-permeable membrane (indicated by the dashed line) from autologous uninfected PBMC devoid of CD4+ T-cells. TLR agonists were added to the culture medium as indicated in the figure. Infected PBMC where the TLR agonists were added to the insert served as positive control for the TLRs' efficient antiviral activity. Notably, CD4+ T-cells do not express TLR7 and 8 and thus do not react to the TLR agonists added. In this setup any inhibitory HIV activity observed when HIV infected CD4+ T-cells were separated by a semi-permeable membrane from the PBMC (devoid of CD4+ T-cells) must be due to factors secreted by the PBMC into the medium, which then diffuse through the semi-permeable membrane (n = 3). This setup with separated cells was compared to PBMC which have been treated identically. Control indicates PBMC infected with HIV. B) Blocking the IFN-α/β receptor (IFN-α/β R) does not reverse the TLR7/8 triggering mediated inhibition of HIV. Neutralizing antibodies to IFN-α/β R was added at 10 µg/ml 30 minutes prior to R-848 to PBMC which was infected two days later with HIV. All values were indicated as relative to the matched control which was arbitrarily set to 1.

When effector cells were separated from target cells by transwells, HIV replication was efficiently inhibited. This finding clearly indicates that soluble factors have a potent anti-HIV effect (Fig. 5). Levels of a large number of cytokines and chemokines were increased after triggering TLR7 and/or 8 (Table 4). Most impressively, IFN-α increased more than 100-fold after triggering either TLR7 and/or 8. The TLR agonists 3M-002 and R-848, which trigger TLR8, caused a more pronounced release of IL-12 and TNF-α than 3M-001. Furthermore, we observed moderate increases of the β-chemokines. Some of those cytokines are known to have anti-HIV activities.

**Table 4:** Induction of cytokines one day after triggering TLR7 and/or 8 in PBMC; indicated in each box upper line are the average±std fold induction# and lower line the corresponding absolute concentrations of cytokines measured in the supernatant [pg/ml] (n = 3) ¶.

	control	3M-001	3M-002	R-848
**IFN-α ¥**	46, 0.1, 0.1¶	1520±1931#	427±738#	1544±1680#
		1000, 370, 84¶	64, 128, 0.1	562, 334, 128
**IFN-γ ¥**	191, 81, 53	0.8±0.2	1.6±1.0	1.3±0.4
		185, 50, 41	507, 50, 79	306, 75, 79
**IL-6 ¥**	3638, 2834, 1555	2.7±0.6	1.7±0.2	2.4±0.3
		7534, 7960, 5174	5731, 5464, 2301	7298, 7168, 3994
**IL-8 ¥**	14813, 14127, 15245	1.0±0.2	0.9±0.1	0.8±0.2
		17587, 15029, 11342	15529, 12319, 11717	13599, 13515, 9427
**MIP-1α ¥**	131, 52, 37	3.0±1.7	1.8±0.4	2.6±0.9
		194, 139, 176	176, 115,72	221, 135, 128
**MIP-1β ¥**	275, 84, 25	5.9±6.2	3.0±1.9	5.1±4.5
		344, 299, 327	394, 208, 129	479, 286,257
**RANTES ¥**	134, 32, 14	7.6±4.1	3.0±0.3	7.0±3.0
		646, 186, 172	356, 102, 42	646, 186, 146
**IL-12 (p40)** *	83, 30, 100	4.6±2.1	43.6±15.0	21.9±9.0
		531, 69, 516	3331, 1803, 3069	1710, 943, 1366
**TNF-α** *	685, 938, 529	1.9±0.6	36.7±12.1	19.0±5.7
		1122, 1449, 1357	30779, 21380, 22470	17560, 14698, 8372

* ¥ IFN-α, IFN-γ, IL-6, IL-8, MIP-1α, MIP-1β, RANTES were measured by enzyme immunoassays, IL-12(p40) and TNF-α were measured with a bead assay (see material and method section).

To assess the significance of the individual cytokines on HIV replication, we have used commercially available neutralizing antibodies to block these cytokines, and followed mainly the providers' instructions in regard to the concentration; in any case we used always concentrations at the upper limit feasible (see material and method section). We have formal proof that the neutralization of IFN-α and TNF-α was effective: the efficacy of the IFN-Rα antibody is shown by the fact that adding this antibody resulted in higher HIV replication in control cultures (Fig. 5B). The TNF-α blocking antibody showed complete suppression of recombinant TNF-α in a transient-transfection assay using a NFκB reporter assay (data not shown). Furthermore, there was a tendency that blocking IL-12, -15 and -18 was also effective in the reversal of TLR7-mediated anti-HIV effects. In any case, neutralization of individual cytokines or of combinations of diverse cytokines was not sufficient to reverse the anti-HIV effects after triggering TLR8 or 7/8 (Table 5). In contrast, the inhibitory activity in cultures treated with the TLR7 agonist was partially reversed when adding nAb against the IFN-Rα, pointing to the known anti-viral role IFN-α has in many settings. To conclude, the enigma concerning the soluble factor responsible for the potent TLR7/8 agonists' anti-HIV effect remains.

**Table 5:** Neutralization of cytokines had no impact on anti-HIV activity subsequent to triggering TLR8 and/or 7/8; indicated are the percent inhibition of anti-HIV activity (median (25 to 75% percentile).

nAb to	3M-001		3M-002		R-848	
	Ø	+	Ø	+	Ø	+
**IFN-γ; (n = 4)**	26.52	5.195	87.28	89.8	81.9	76.39
	(13.92 to 55.05)	(−53.89 to 43.42)	(80.8 to 92.15)	(66.68 to 93.46)	(73.03 to 90.22)	(69.78 to 86.03)
**IFN-αR; (n = 6)**	14.08	67.4#	83.75	90.42	91	91.15
	(−5.85 to 48.7)	(11.54 to 82.79)	(68.43 to 91.81)	(77.7 to 97)	(69.49 to 91.81)	(76.16 to 95.41)
**IFN-αR/IL-12; (n = 3)**	7.16	3.43	89.65	68.86	94.63	80.24
	(−18.86 to 12.42)	(−17.64 to 14.28)	(77.85 to 93.29)	(−17.63 to 96.64)	(93.46 to 94.87)	(51.99 to 96.38)
**IL-12; (n = 6)**	39.22	59.58	78.95	84.6	78.02	82.77
	(−35.8 to 65.03)	(12.13 to 71.96)	(66.53 to 88.95)	(72.83 to 92.96)	(74.74 to 93.71)	(79.58 to 94.72)
**IL-15; (n = 19)**	57.3	25.9	77.1	80.5	77.3	78.4
	(−0.7 to 62.3)	(−74.5 to 71.8)	(70.3 to 81.8)	(67.7 to 85.2)	(70 to 83.9)	(69 to 83)
**IL-12/IL-15/IL-18; (n = 6)**	39.22	63.46	78.95	81.14	78.02	81.16
	(−35.8 to 65.03)	(21.24 to76.5)	(66.53 to 88.95)	(67.77 to 93.14	(74.74 to 93.71)	(75.65 to 94.72)
**IL-12/IL-15; (n = 6)**	39.22	66.94	78.95	87.51	78.02	86.27
	(−35.8 to 65.03)	(7.645 to 80.42)	(66.53 to 88.95)	(80.7 to 94.21)	(74.74 to 93.71)	(80.19 to 95.8)
**IL-12/IL-18; (n = 6)**	39.22	63.58	78.95	77.92	78.02	79.87
	(−35.8 to 65.03)	(17.71 to 73.26)	(66.53 to 88.95	(54.59 to 92.16)	(74.74 to 93.71)	(69.7 to 93.25)
**IL-15/IL-18; (n = 6)**	39.22	59.17	78.95	77.4	78.02	72.14
	(−35.8 to 65.03)	(2.365 to 66.42)	(66.53 to 88.95)	(51.38 to 92.02)	(74.74 to 93.71	(52.17 to 91.98)
**IL-18; (n = 6)**	39.22	64.95	78.95	80.56	78.02	78.81
	(−35.8 to 65.03)	(0.275 to 79.42)	(66.53 to 88.95)	(63.29 to 93.67)	(74.74 to 93.71)	(66.42 to 94.34)
**MIP-1α; (n = 4)**	43.91	38.39	87.28	86.37	81.9	81.83
	(13.91 to 72.56)	(14.07 to 66.64)	(80.8 to 92.15)	(81.85 to 91.57)	(73.03 to 90.22)	(69.36 to 89.27)
**MIP-1β; (n = 4–6)**	26.39	36.17	89.79	74.64	81.9	65.26
	(13.91 to 54.92)	(−18.75 to 64.47)	(80.8 to 93.78)	(−12.02 to 88.91)	(73.03 to 90.22)	(49.54 to 82.02)
**RANTES; (n = 4)**	26.39	10.73	87.28	87.4	81.9	77.94
	(13.91 to −54.56)	(−125.1 to 66.67)	(80.8 to 92.15)	(69.98 to 91.5)	(73.03 to 90.22)	(63.78 to 90.21)
**SDF-1; (n = 4–6)**	35.56	−8.055	87.28	89.64	81.9	79.11
	(13.91 to 55.4)	(−144.1 to 48.93)	(80.8 to 92.15)	(66.33 to 93.3)	(73.03 to 90.22)	(59.21 to 88.18)

# P = 0.012 (paired T-test)

### HIV replication is blocked after entry but before integration

**Figure 6:**
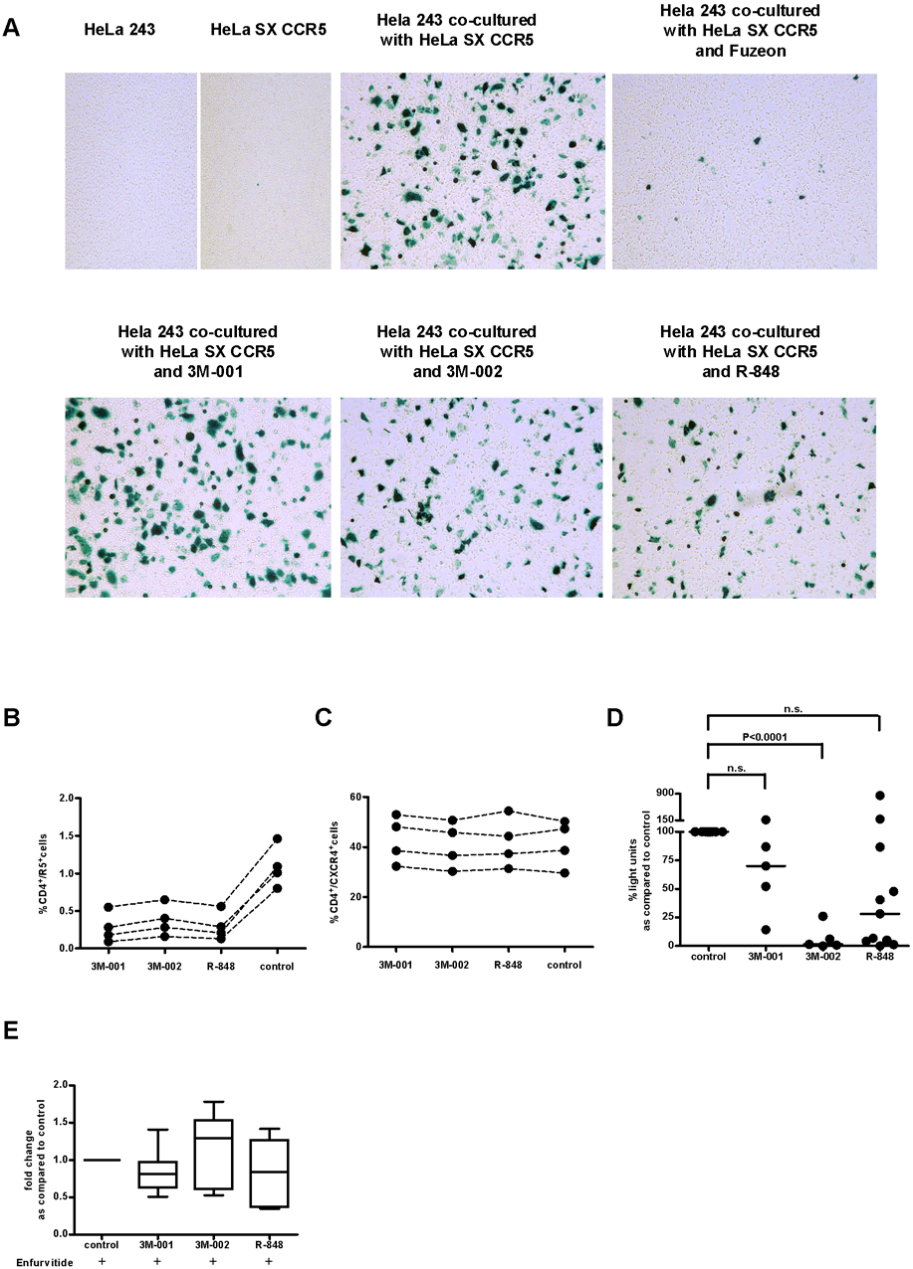
Triggering TLR8 and/or 7/8 blocks HIV replication after HIV entry but before integration. (A) Unspecific inhibitory effects of the compounds triggering TLR7 and/or 8 on HIV entry was examined with a cell-cell-based fusion assay, in which one cell line expresses the HIV-gp120 and an LTR-driven *lacZ* gene (HeLa SX CCR5) and the other cell line expresses the HIV receptor complex (CD4 and CCR5) and Tat (HeLa 243). Fusion of these cell lines will result in Tat driven LTR *lacZ* gene expression which we quantified histochemically by staining for β-galactosidase activity (n = 2). (B and C) Percentage of C4+ T-cells expressing CCR5 and CXCR4 was quantified by flow-cytometry two days after triggering TLR7 and/or 8 of PBMC Dashed lines indicate the matched samples. (D) Replication incompetent lentiviral viruses pseudotyped with VSV envelope which encodes a luciferase reporter gene were used to assess whether TLR7 and/or 8 triggering affects the early step in the replication cycle of HIV. Notably VSV uses a totally different entry mechanism than HIV which is highly unlikely to be affected by the TLR7 and/or 8 agonists. (E) Assessment whether TLR agonists have any effects on HIV replication at the transcriptional level or later in the HIV replication cycle. For this purpose, CD4+ T-cells were infected with HIV and spreading infection was blocked by adding the fusion inhibitor enfurvitide. These CD4+ T-cells with integrated HIV were co-cultured with either autologous PBMC devoid of CD4+ T-cells previously stimulated by TLR7 and/or 8 agonists vs unstimulated autologous PBMC devoid of CD4+ T-cells. Thus, any change in HIV output would be due to the effects on either transcription or later events in the HIV replication cycle due to TLR7 and/or 8 triggering (analysis of variance: n.s.; n = 6).

For simplicity, HIV replication can be divided into entry, post-entry, and post-integration steps. Using a cell-cell-based fusion assay in which one cell line expresses CD4 and CCR5 and another cell line expresses CCR5-tropic gp120, we have not observed any non-specific blocking of HIV fusion from the compounds examined (Fig. 6A). Notably, HeLa cells do not express TLR7 or 8.

Expression level of the HIV receptor complex is required for HIV to enter a cell. Indeed, we found that triggering TLR8 and/or 7/8 resulted in a decrease in the percentage of CD4+ cells expressing CCR5 while the percentage of CD4+ cells expressing CXCR4 was unchanged (Fig. 6B). The median intensity of CCR5 and CXCR4 was not affected by triggering TLR7 and/or 8 (data not shown). The decrease of CCR5 may be explained by the increased release of β-chemokines. However, X4-tropic HIV strains were inhibited to the same extent as R5-tropic strains. Thus, other anti-HIV active mechanisms must be blocking HIV replication. Additionally, we used a lentiviral based reporter virus pseudotyped with the envelope from vesicular stomatitis virus. This virus uses an entirely different entry mechanism than HIV and was inhibited to the same extent as HIV when the tissue was treated with compounds triggering either TLR7 and/or TLR7/8 (Fig. 6D).

We further wanted to substantiate the above-mentioned findings using an experimental set-up that examines whether the TLR7/8 agonists induced effects inhibits HIV replication at the level of transcription or later in the HIV replication cycle. For this purpose, we infected isolated CD4+ T-cells with HIV using spinoculation [21] and added 12 h later the fusion inhibitor enfurvitide which prevents spreading infection. Spinoculation was used to increase the number of HIV infected CD4+ T-cells which will increase the sensitivity of the read out. Thereafter, we co-cultured these infected CD4+ T-cells with autologous PBMC devoid of CD4+ T-cells which had been previously exposed to the TLR7/8 agonists. In this setup, the resulting HIV production was solely due to the ongoing HIV transcription of the CD4+ T-cells being already infected. If the changes in the microenvironment induced had any effect on HIV replication after integration, we would see a clear decrease of resulting HIV replication in samples treated with the TLR agonists. However, this was not the case (Fig. 6E), indicating that they display their inhibitory activity prior to integration of HIV into the host chromosome.

## Discussion

We previously showed that triggering TLR7/8 causes changes to the lymphoid microenvironment that are unfavorable for HIV replication [12]. Here we sought to identify the individual contributions of TLR7 and 8 to the overall anti-HIV activity and to gain insights into the mechanisms of how TLR trigger anti-viral defenses. We found that (i) the TLR7- or 8-mediated anti-HIV activity depended on the origin of the lymphoid tissue, (ii) dendritic cells are indispensable for the anti-HIV effect, (iii) the main effector cells for the anti-HIV activity were NK-cells and CD8+ T-cells, and iv) soluble factors mediated the anti-HIV effects and were not reversed when the antiviral active cytokines IFN-α, -γ, or TNF-α were neutralized.

The TLR7/8 agonist R-848 shows strong anti-HIV activity in PBMC and lymphoid suspension cells from tonsils [12]. In this study, we used two agonists which selectively trigger either TLR7 or 8 [22]. Their potency depended strongly on the origin of lymphoid tissue. Triggering TLR7 was primarily responsible for the anti-HIV activity in tonsillar lymphoid suspension cells, but it had only limited and inconsistent effects in PBMC. In contrast, triggering TLR8 had strong anti-HIV activity in PBMC; it had also consistent but somewhat less anti-HIV activity in tonsillar lymphoid suspension cells. Because of limited availability of tonsillar tissue, we focused in most experiments on PBMC for elucidating the anti-HIV mechanism(s) of TLR7 and/or 8 triggering.

Based on the sentinel function and the expression of TLR8, we speculated that MDC initiate the complex changes in PBMC that are eventually unfavorable for HIV replication. Indeed, when we depleted DC and DC precursors from PBMC, the anti-HIV effect from TLR7/8 triggering was mostly lost. Not surprisingly, depletion of these cell subsets from untreated cultures resulted in modest increases of HIV replication, pointing to HIV's ability to trigger TLR directly [5], [23], [24] and thus to initiate an anti-viral defense program, although less potently than the TLR agonists examined. The potent antiviral defense program initiated by TLR8 or 7/8 was also reflected when exposing MoMDC to the TLR agonists: they showed cell clumping, up-regulation of the cell surface marker CD80, CD83 and CD86 as well as pronounced secretion of a number of cytokines. Triggering solely TLR7 had no visible effect on MoMDC.

Previously, we reported that depletion of B cells partially reversed the anti-HIV activity of R-848 [12]. Strikingly, when we depleted NK and CD8+ T-cells prior to adding the TLR8 or 7/8 agonists to PBMC, we observed a rather complete reversal of the anti-HIV effects; depleting either NK or CD8+ T-cells showed only partial inhibition, suggesting that there is an additive effect when stimulating NK and CD8+ T-cells or there is an eminent cross-talk between these cell-populations necessary for this striking anti-HIV effect. Triggering TLR7 in PBMC depleted from NK and CD8+ T-cells showed inconsistent effects which reflect its overall inconsistent anti-HIV effects. Since NK and CD8+ T-cells are the key effector cells for the TLR8-mediated HIV inhibitory activity, their low or even absent numbers in tonsillar lymphoid suspension cultures may explain the lower TLR8 agonist's anti-HIV effects in that tissue as compared to PBMC. The robust HIV inhibitory activity in tonsillar lymphoid tissue and the rather poor one in PBMC in response to TLR7 triggering speak in favor of additional tissue type specific mechanism(s) beyond NK and CD8+ T-cells which may explain the differences between TLR7 and TLR8 agonists. Notably, while we have not assessed functional differences, the percentages of DC are similar in the lymphoid tissue studied.

The potent cell activation subsequent to triggering TLR8 or TLR7/8 is reflected by an up-regulation of intracellular IFN-γ and TNF-α and increased cytolytic activity of NK and CD8+ T-cells. Surprisingly, levels of expression of inhibitory (CD94, KIR2DL2, KIR3DL1, ILT2) and activating receptors (NKp30, NKp46, NKp44, DNAM-1) on NK cells were similar between the controls and PBMC treated with TLR agonists (data not shown).

Since NK and CD8+ T-cells do not respond directly to TLR7/8 agonists [25], their activation has to occur either by cell-cell contact with a TLR7/8-responsive cell or by soluble factors. The most likely candidates are IL-12, -15, and -18, which are released from DC upon their activation. However, blocking the individual cytokines or all of them with neutralizing antibodies did not reverse the anti-HIV activity and neither prevented the cellular activation of NK and CD8+ T-cells that is consistent with a recently published study [26]. Thus, we assume that cell-cell contact and/or a variety of soluble redundant factors will ultimately result in the activation of these effector cells. Indeed, the significance of bi-directional interactions between DC and NK cells has been convincingly demonstrated for an efficient immune defense [17], [18].

So how do NK and CD8+ T-cells block HIV infection? To answer this question, we used trans-well experiments, in which HIV-infected CD4+ T-cells were separated from PBMC devoid of CD4+ T-cells. We found that separating the effector from the target cells did not abrogate the anti-HIV effects, thus implicating soluble factors in the process. Indeed, triggering TLR7 and/or 8 resulted in prominent changes in the cytokine profile. However, we were not able to assign the anti-HIV effect to one distinct cytokine even though we neutralized a large number of soluble factors either alone or in combination. The lack of any reversal of the antiviral activity subsequent to triggering TLR8 or TLR7/8 when IFN-α was neutralized is most puzzling and speaks in favor of very potent unknown factor(s) inhibiting HIV. In contrast, the inhibitory activity in response to TLR7 triggering was partially reversed when IFN-α was neutralized: this indicates that these above postulated factor(s) are not secreted or not to the same amount when exposing lymphoid tissue to TLR7 agonists. While not statistically significant, it appeared that blocking IL-12, -15 and -18 resulted also in some reversal of the anti-HIV effects in the case of triggering TLR7. We assume that TLR7 triggering activates DC which in turn releases IL-12, -15 and -18 critical for activating downstream effector cells such as NK or CD8+ T-cells.

While the data with the trans-wells were unambiguous, cell-cell contact may substantiate the anti-HIV effects. However, NK cells showed only minor changes of activating and/or inhibiting receptors (see above) and thus, we do not assume that they play a major role. In contrast, TRAIL showed a prominent up-regulation on NK cells. This increased TRAIL expression is reminiscent of a recent report describing the TLR7 triggering dependent generation of killer PDC expressing TRAIL able to induce apoptosis of HIV-infected SupT1 cells [27]. The potential effects of TRAIL may be masked by the potent inhibitory anti-HIV effects of soluble factors in our experimental set-up that may explain the lack of any reversal of anti-HIV activity when neutralizing TRAIL (data not shown). The potent HIV-inhibition by soluble factors is reminiscent of the long-searched CD8+ T-cell antiviral factor, CAF [28].

Triggering TLR7/8 appears to inhibit HIV before its integration into the host-genome. Downregulation of CCR5 after release of β-chemokines may contribute to the anti-HIV activity when challenging the lymphoid tissue with CCR5-tropic strains. However, other anti-HIV mechanism(s) must be involved. Down-regulation of CCR5 was not complete, and more importantly, X4-tropic strains were also inhibited. We excluded an unspecific effect on the fusion process of the TLR agonists as examined by a cell-cell based fusion assay. Furthermore, we used replication-incompetent viruses pseudotyped with VSV envelope, which enter cells by an entirely different mechanism(s) than HIV. We observed a similar inhibition of the reporter gene in tissue exposed to TLR agonists, also suggesting that these agonists act after entry but before integration/transcription. To substantiate this finding, we investigated whether the TLR agonists induced changes influence HIV replication at the level of transcription: for that purpose CD4+ T-cells were infected overnight and spreading infection was inhibited by adding the fusion inhibitor enfurvitide. The infected CD4+ T-cells were subsequently co-cultured with previously TLR7/8 agonist stimulated autologous PBMC devoid of CD4+ T-cells. Thus, resulting HIV output from this co-culture reflects exclusively HIV transcription from HIV-infected CD4+ T-cells. In this setup, the TLR agonists had no inhibitory activity as compared to the control excluding any inhibitory action at the transcriptional level.

In summary, we propose a new model, in which triggering TLR7 and/or 8 of DC activates NK cells and CD8+ T-cells. These cells, in turn, induce the release of soluble factors that result in a microenvironment unfavorable to HIV infection. Since B cells express TLR7, they may be activated directly or indirectly by the corresponding trigger, but in any case, they have less anti-HIV activity than NK and CD8+ T-cells. The inability to identify a specific soluble factor is characteristic of the vast array of changes induced by triggering pattern recognition receptors and points to the poorly understood redundancy of the innate immune responses. As outlined in the introduction, we would like to reiterate the fact that compounds triggering TLR7 and/or 8 also strengthen the adaptive immune responses. Thus, these compounds may combine by acting on diverse arms of the immune response optimal properties as immunomodulatory drugs in HIV infection.

## Materials and Methods

### Primary lymphoid tissue, cell lines, separation of cells, and generation of monocyte-derived myeloid dendritic cells

PBMC obtained from the blood bank (Stiftung Zürcher Blutspendedienst SRK, Zürich, Switzerland) were prepared by Ficoll Hypaque and cultured in RPMI (BioWhittaker, Verviers, Belgium) supplemented with 10% fetal calf serum (PAA Laboratories, Vienna, Austria), 10 U/ml IL-2 (National Institutes of Health, AIDS Repository), 2 mM L-glutamine (Invitrogen Life Technologies, Paisley, Scotland), and 1% penicillin/streptomycin (Invitrogen Life Technologies). We were authorized by the Ethical Committee of the University Hospital Zurich to receive tonsillar tissue from the Department of Otorhinolaryngology, University Hospital of Zurich. Tonsillar tissue was minced and subsequently put into a cell strainer (70 µm; BD Biosciences, San Jose, CA) where the tissue was grinded through the sieve with a syringe plunger. Erythrocytes were lysed with ACK cell lysing buffer (Cambrex, Walkersville, MD). Lymphoid cells were washed. These lymphoid suspension cells were cultured in RPMI containing 15% FCS, 1% penicillin/streptomycin, 2.5 µg/ml Fungizone, 2 mM L-glutamine, 1 mM sodium pyruvate, and 1% nonessential amino acids. HeLa (ATCC #CCL 2) and 293 T (ATCC #CRL 1573) cells were cultured in DMEM (10% FCS, 1% penicillin/streptomycin), EGFP-K562 [29] cells in RPMI resp. supplemented with 10% FCS, 2mM glutamine and 1% penicillin/streptomycin. G418 was in addition added to the RPMI as antibiotic selection for EGFP-K562 cells for guaranteeing transgene EGFP expression.

To deplete PBMC from a distinct cellular subset, we used MicroBeads from MACS Miltenyi (Bergisch Gladbach, Germany). Specifically we used CD56 MicroBeads for NK cells, CD4 MicroBeads for CD4+ T-cells and CD8 MicroBeads for CD8+ T-cells. To sort for PBMC lacking dendritic cells (DC) or their precursors, PBMC were stained with antibodies to the lineage markers CD3, CD19, CD56 and CD14, labeled to FITC, and to the cell-surface markers HLA-DR and CD16, and subsequently sorted for the lineage-positive but CD16-negative cell population on a FACS Aria as previously described [30]. Efficacy of depletion was always >95% as verified by flow cytometry.

To generate monocyte-derived myeloid dendritic cells (MoMDC), we cultured monocytes which were isolated using CD14 microbeads in RPMI supplemented with 1000 U/ml each of GM-CSF and IL-4 for 6 days. IL-4 and GM-CSF were purchased from ImmunoTools (www.immunotools.de). The MoMDC had a purity of >93% as defined by the cell surface expression of CD11c using flow-cytometry and were cultured at a density of 1×10^6^/ml.

### Reagents and antibodies for neutralization of cytokines

The TLR ligands 3M-001, 3M-002, and R-848 were generously provided by 3M Pharmaceuticals (St. Paul, Minnesota). The following antibodies were used: mAb anti-human interferon-α/β receptor chain 2 at 10 µg/ml (clone MMHAR-2, PBL Biomedical Laboratories, purchased from Alexis, Lausen, Switzerland), anti-human IL-18 at 100 ng/ml (clone 125-2H; MBL Medical & Biological Laboratories), anti-human IL-15 at 500 ng/ml (clone 34559; R&D), anti-human IL-12 at 20 ng/ml (ab9992, Abcam, Cambridge, UK), anti-human MIP-1α at 4 µg/ml (ab10381 Abcam), anti-human MIP-1β at 3 µg/ml (#PP1051, Acris, Hiddenhausen, Germany), anti-human Rantes at 7 µg/ml (ab9679, Abcam), anti-human IFN-γ at 100 ng/ml (ab9657, Abcam) and anti-human SDF-1α at 5 µg/ml (#PP1067, Acris, Hiddenhausen, Germany).

### Colometric viability assay

The Colorimetric Cell Viability WST-1 kit (Roche, Mannheim, Germany, Cat. No 1 664 807) is based on the metabolism of the tetrazolium salts to water-soluble, orange formazan dyes by dehydrogenases present in viable cells. The WST-1 kit was used according to the instruction of the provider.

### HIV strains

We used the prototype HIV strains NL4-3, 49.5, JR-CSF and 89.6; all were proviral DNAs. To generate viral stocks, 293 T cells were transfected with proviral DNA by polyethylenimine (PEI), and supernatants were harvested 2 days later, passed through a 0.22-µM filter, and stored at -80°C until use.

### Infectivity assays

PBMC or tonsillar lymphoid suspensions cells were cultured in wells of 96-well plates at a concentration of 10×10^6^ cells/ml, pretreated for 2 days with the corresponding TLR agonist, according to the specific aim of the experiment, and subsequently infected overnight with 1–3 ng HIV/well. Cultures were extensively washed, and new medium was added with the corresponding agonists. HIV replication was monitored by quantifying p24 over time by an in-house p24 ELISA [31].

When we assessed the role of distinct cytokines for their significance in blocking HIV replication, we added the neutralizing antibodies (concentrations see above) 30 minutes prior to the TLR7/8 agonists.

Effects of TLR ligands on transcription or later stages of HIV replication were examined in a co-culture assay where HIV-infected CD4+ T-cells were cultured with autologous PBMC depleted of CD4+ T-cells. The CD4+ T-cells were infected by spinoculation with HIV [21] and kept overnight. The autologous PBMC depleted of CD4+ T-cells were activated prior to the co-culture by the TLR7 and/or 8 agonists. We added the fusion inhibitor enfurvitide at 50 µg/ml to the co-culture in order to prevent spreading infection. Thus, this experimental set-up permits to assess the effects of the TLR7 and/or 8 agonists on HIV replication at the level of HIV transcription or later in the HIV replication cycle.

For inter-donor comparisons, we expressed the p24 values over time as the area under the curve (AUC) representing HIV replication. We calculated the percent inhibition of replication by any given drug and concentration by first expressing the AUC of treated cultures as a percentage of an untreated, infected control culture and then subtracting the percent of AUC treated samples from 100%. In some experiments, we also expressed effects of TLR ligands on HIV replication as fold change compared to the control.

### NK-cytotoxicity assay

PBMC treated with the compounds that trigger TLR7 and/or 8 were co-cultured with the NK cell–responsive cell line EGFP-K562 [29] at an effector : target ratio of 1:10 for 6 h. The expression of enhanced green fluorescent protein (EGFP) allowed us to precisely quantify the number of residual EGFP-K562 cells. NK cytotoxic activity was calculated by expressing the residual EGFP-K562 cells as a percentage of the initial number of target cells added to the co-culture.

### Flow cytometry

Monoclonal antibodies (mAb) to the following markers were used: IFN-γ (554552), TNF-α (559321), CD107a (555801), CD4 (555346), CD8 (555634), CD69 (555533), CD56 (555518 and 345811), CD80 (557227), CD83 (556855), CD86 (555658), CD226 (559789), CD336 (558563), CXCR4 (555974), CCR5 (556903), CD3 (555339), CD19 (555412), CD14 (555397), HLA-DR (340549), CD20 (559776), CD11c (333144) and CD123 (340545) (all from BD Pharmingen), CD16 (130-091-246; Miltenyi), NKp30 and NKp46 (Beckman Coulter, Krefeld, Germany), NKG2D (R&D Systems, Abingdon, UK), and CD94 (provided by L. Lanier, University of California, San Francisco, CA). Quantification of DC was done by staining PBMC with the lineage kit (Lin1 FITC 340546) and mAB against CD11c, CD123 and HLA-DR. To stain for intracellular cytokines, we used the Cytofix/Cytoperm Kit (BD, #554714), according to the manufacturer's instructions. Staining for CD107a was done by incubating TLR7- and/or 8-treated PBMC with a mAb to CD107a and Brefeldin A (BD Pharmingen, Allschwil, Switzerland) for 5 h. Data were acquired on a FACS Calibur and analyzed by FlowJo software (TreeStar)).

### Transwell experiments

To determine if cell-cell contact or soluble factors are involved in the anti-HIV effects after TLR triggering, HIV-infected CD4+ T-cells were cultured at the bottom of a well of a six-well plate, and the PBMC devoid of CD4+ T-cells in the insert. As controls served untreated PBMC infected with HIV as well as PBMC infected similarly as the CD4+ T-cells which were placed at the bottom of wells and the TLR agonists added to the inserts. Inserts with a pore size of 4 μ were purchased from Millipore. The compounds triggering TLR were added to the medium. CD4+ T-cells do not respond to TLR7/8 since they have no TLR7/8. Thus, any observed effects must be due to soluble factors released from the treated PBMC devoid of CD4+ T-cells.

### Cytokine measurement

In the initial experiments, IL-6 (D6050), -8 (D8050), -12 (D1200), TNF-α (HSTA50), RANTES (DRN00B), MIP-1α (DMA00), and -β (DMB00) were quantified by enzyme immunoassays (Quantikine ELISA, R&D Systems), according to the manufacturer's instructions, IFN-α (Bender Med Systems BMS216) and -γ (Hycult Biotechnology, Hbt human Interferon gamma, HK030S) were quantified by enzyme immunoassays from Bender MedSystems Diagnostics and HyCult Biotechnology, respectively. We then changed to multiplexed particle-based flow cytometric cytokine assay for measuring cytokines [32]. Bioplex cytokine kits were purchased from BioRad (Ismaning, Germany). The procedures closely followed the manufacturer's instructions. The analysis was conducted using a conventional flow cytometer (FC500 MPL, BeckmanCoulter, Nyon, Switzerland). For each data set, we have indicated what method was used.

### Cell-based fusion assay

A cell-based fusion assay consisted of HeLa cells that express gp140 from LAI and the HIV transactivator tat (HeLa-Env/LAI) [33] and HeLa cells that express CD4 and the LTR-driven *lacZ* gene (HeLa SX CCR5) [34]. Fusion of HeLa-Env/LAI cells and HeLa SX CCR5 results in transcription of the *lacZ* gene. The extent of fusion was quantified by assaying β-galactosidase activity in cell lysates (E2000; Promega, Wallisellen, Switzerland) or by histochemical staining of cells for β-galactosidase activity (Roche Molecular Biochemicals), according to the manufacturer's instructions. HeLa-Env/LAI cells were treated with the various compounds and the HIV fusion inhibitor enfurvitide [35]. Subsequently, a similar number of HeLa SX CCR5 cells were added to allow fusion to take place. β-Galactosidase activity was assessed 12 h later.

### Pseudotype virus preparation and challenge of cells with pseudotype viruses

To prepare HIV pseudotype virus packaged by vesicular stomatitis virus (VSV) Env, an HIV proviral construct encoding a luciferase reporter gene (pNL4-3.Luc.R^−^E^−^) was cotransfected with a VSV Env expression vector in 293T cells as described [36]. To determine permissiveness to entry by pseudotype viruses, PBMC cultures were pretreated with R-848 for 1 day, exposed to pseudotype virus overnight, and cultured for additional 2 days before analysis. Luciferase expression was quantified with the luciferase assay system from Promega.
